# Interethnic Variations and Clinical Features of Spondyloarthropathies in a Middle Eastern Country

**DOI:** 10.2174/1874312901812010029

**Published:** 2018-03-07

**Authors:** Mohammed Kamil Quraishi, Humeira Badsha, Bhavna Khan, Muhammad Shahzeb, Srilakshmi Hegde, Ayman Mofti, Kong Kok Ooi

**Affiliations:** 1 Department of Urology, Medway Maritime Hospital, Kent, England, UK; 2 Department of Rheumatology. Dr. Humeira Badsha Medical Center, Dubai, UAE.; 3 Integrated Rheumatology and Arthritis Centre, Dubai Healthcare City, Dubai.; 4 Department of Medicine, Jinnah Medical College Hospital, Karachi, Pakistan.; 5 Department of Rheumatology, Al Biraa Arthritis & Bone Center, Dubai, UAE.; 6 Department of Rheumatology, Tan Tock Seng Hospital, Singapore

## Interethnic Variations and Clinical Features of Spondyloarthropathies in a Middle Eastern Country

The Open Rheumatology Journal, **2018**, 12: 10-18

The correct address of corresponding author which is mentioned below:

***Department of Urology, Medway Maritime Hospital, Windmill Road, Gillingham, Kent, UK ME7 5NY. Email: MKQURAISHI@doctors.org.uk***

The original address provided was: 

***Department of Urology, Medway Maritime Hospital, 5 Miraj Avenue, Sparkhill, Birmingham, West Midlands, UK, B11 4JW; E-mail: MKQURAISHI@doctors.org.uk***

The correct affiliation of Dr. Bhavna which is mentioned below:

***Integrated Rheumatology and Arthritis Centre, Dubai Healthcare City, Dubai***

The original affiliation provided was: 

***Department of Rheumatology, Dubai Mall Medical Center, Dubai, UAE***

